# Prognostic Value of High-Sensitivity C-Reactive Protein in In-Stent Restenosis: A Meta-Analysis of Clinical Trials

**DOI:** 10.3390/jcdd9080247

**Published:** 2022-08-04

**Authors:** Ming Yi, Lu Wu, Xiao Ke

**Affiliations:** 1 Department of Cardiology, Liuyang Hospital of Traditional Chinese Medicine, Liuyang 410300, China; 2 Department of Clinical Medicine, University of South China, Hengyang 421001, China; 3 Department of Cardiology, Fuwai Hospital, Chinese Academy of Medical Sciences, Shenzhen, (Shenzhen Sun Yat-sen Cardiovascular Hospital), Shenzhen 518057, China

**Keywords:** high-sensitivity C-reactive protein, in-stent restenosis, meta-analysis

## Abstract

Background: A risk assessment of in-stent restenosis (ISR) patients is critical for providing adequate treatment. Nevertheless, the prognostic value of high-sensitivity CRP (hs-CRP) levels on ISR has not been consistently demonstrated in clinical studies. In the current meta-analysis, we aim to assess the predictive role of hs-CRP in patients treated with stenting. Methods: We searched PubMed, Web of Science, Embase, and the Cochrane Registry through May 2022. We selected random control trials that compared the effects of different interventions, and that revealed the effects of hs-CRP. Two reviewers independently screened the articles, extracted the data, and assessed the quality of the studies according to the PRISMA guidelines (Preferred Reporting Items for Systematic Reviews and Meta-Analyses). The data were pooled using a random-effects meta-analysis. Results: Nine articles were included in the meta-analysis. A total of 1.049 patients received stent implantation, and 185 ISR events were recorded during the 1–12-month follow-up period. Baseline hs-CRP levels were not associated with the prediction of ISR among patients receiving stent implantation. The OR of hs-CRP for ISR was 1.81 (0.92–2.69). In the subgroup analysis, 6–12-month hs-CRP levels, diabetes mellitus (DM), and age ≥60(years)were associated with a higher risk of ISR. Conclusions: This meta-analysis shows that higher levels of baseline hs-CRP are not associated with an increased risk of ISR in stented patients. However, an increased risk of ISR was associated with hs-CRP levels at 6 to 12 months of follow-up, which is higher in studies with diabetes mellitus patients and the elderly.

## 1. Introduction

Percutaneous coronary intervention (PCI) is one of the most important treatments for atherosclerotic cardiovascular disease (ASCVD) patients [1]. Prompt revascularization can save patients’ lives and improve their prognosis. However, problems with in-stent restenosis (ISR) during follow-up diminish this benefit [2]. It is a fact that the clinical use of drug-eluting stents (DESs) has significantly reduced the incidence of ISR compared to bare-metal stents (BMS) [3]. On the other hand, the incidence rate for patients receiving DES implantation is still 5% to 10% [4]. Moreover, cumulative stenting is becoming common given the dramatic increase in the number of ASCVD patients [5]. ISR remains a significant limiting factor for PCI.

The identification and control of risk factors are critical to improving the diagnostic and treatment capabilities of ISR as well as prophylactic programs [6]. Our previous research implies that the risk factors for DES-ISR and BMS-ISR are basically the same, with age and diabetes being the most important factors of concern [7]. Additionally, a considerable amount of literature has identified numerous risk factors that are known to play a role in determining progress in ISR [8,9,10]. Those factors can be briefly distinguished by inflammatory markers, operational technical characteristics, and coronary lesion characteristics. One of the most common causes of ISR is vascular inflammation associated with atherosclerosis [11,12]. In such cases, inflammatory biomarkers are usually used to help detect ISR and monitor its assessment, prognosis, and treatment delivery. Since 2010, the plasma concentration of hs-CRP (high-sensitivity CRP) has been used as a biomarker for disease prognosis in patients with ASCVD [13]. It could also be useful to establish a high concentration limit for hs-CRP that could be used by physicians for the diagnosis of acute myocardial infarction [14] and to predict cardiovascular events [15]. Hs-CRP is more suitable than CRP in terms of risk stratification in vulnerable patient populations, and hs-CRP can partially replace CRP in clinical use [16]. The advantages of hs-CRP over CRP appear to be more clear in chronic inflammation statues [17]. The end cost/effectiveness of hs-CRP screening is still an area of controversy, but making physicians aware of the positive relationship between high hs-CRP and ASCVD is a priority to improve the median survival and life quality of patients [18]. Some previous studies have pointed out that hs-CRP is an independent risk factor for ISR [19], but some studies have reached opposite conclusions. Additionally, baseline hs-CRP did not indicate more severe stenosis [7]. However, there is, to date, only one systematic review of the clinical trials validating hs-CRP and ISR. Therefore, we aim to investigate the prognostic value of hs-CRP to ISR.

Drugs, new-generation DESs, and drug-coated balloons were used to treat ISR and reduce serum hs-CRP, therefore possibly contributing to its therapeutic effects [20]. Gaztanaga J [21], Rosa W C [22], and Yao W [23] revealed the anti-inflammation effects of oral medications on ISR outcomes. Jung J H [24], Kochiadakis G E [25], and Wang J [26] evaluated the effects of modified stents on variations in ISR outcomes and plasma hs-CRP levels. Hs-CRP screening has become a cost/effectiveness indicator of clinical trials [19]. A previous systematic review conducted by Zhu X et al. [19] only included retrospective studies, and multiple factors (e.g., age, diabetes, and follow-up times) were not adequately analysed. In order to obtain a clear answer, we re-examined the literature based on randomised controlled clinical trials (or prospective cohort studies specifically evaluating the diagnostic value of hs-CRP) to assess the diagnostic prediction value of hs-CRP in patients undergoing stenting.

## 2. Materials and Methods

This systematic review and meta-analysis were conducted in accordance with the PRISMA statement and was registered with INPLASY (registration URL: https://inplasy.com accessed on 31 May 2022, ID: INPLASY202250170, DOI: 10.37766/inplasy2022.5.0170).

### 2.1. Search Strategy

**Figure 1:**
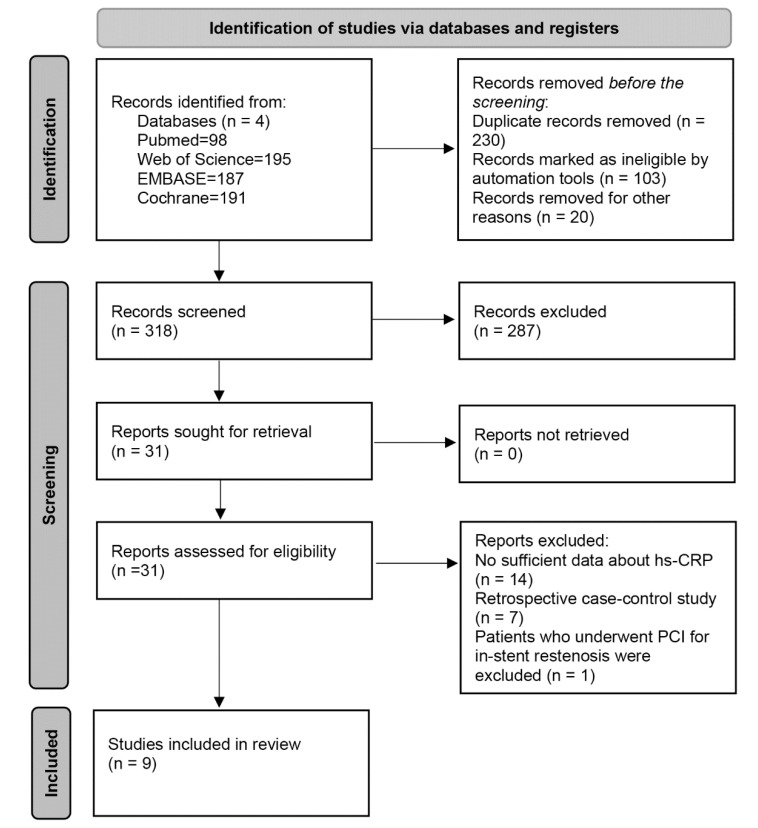
Flow diagram of eligible studies included in the meta-analysis.

A search strategy for the entire study was developed by Ke Xiao, and two authors (Yi Ming and Lu Wu) independently searched the PubMed, Web of Science, Embase, and Cochrane databases to identify relevant studies that had been published as of March 2022. The search keywords were “Percutaneous coronary interventional“, “in-stent restenosis”, and “high-sensitivity C-reactive protein”. The post-search literature was screened, and references to the included studies were manually searched for additional relevant publications. The specific literature search process is shown in Figure 1.

### 2.2. Inclusion Criteria

Studies were included if they met the following criteria: randomised controlled studies or prospective cohorts clearly stating that hs-CRP was one of the main exposure factors for ISR in patients undergoing PCI and a 95% confidence interval (CI) provided for the odds ratio (OR) for mortality or for the reported case data for the intervention, and the use of control groups to calculate these parameters.

### 2.3. Endpoint

The primary endpoint of this systematic review was in-stent stenosis. Restenosis was detected using coronary angiography [6], and higher hs-CRP levels were a positive predictor of ISR events. Because the RCTs did not report ISR as a single endpoint, manual retrieval of the incidence of ISR among composite endpoints was required. In addition, prospective cohort studies with ISR as the target endpoint to evaluate the diagnostic predictive value of hs-CRP were also included.

### 2.4. Quality Assessment

The Revman tool was used to assess the study quality of all of the included full-text articles. We looked at (1) random sequence generation, (2) allocation concealment, (3) participant and personnel blinding, (4) outcome assessment blinding, (5) incomplete outcome data, (6) selective reporting, and (7) other potential sources of bias.

### 2.5. Data Extraction

Yi Ming and Lu Wu extracted data from the included articles independently using electronically extracted files based on predefined standardisations. The following information was extracted from each of the articles used in the final review: investigator, year of study, study design, number of participants, mean age, type of treatment, duration of follow-up, biomarker testing method, whether diabetes was reported as a comorbidity, OR and concomitant 95% confidence intervals, and information on the cases reported in the intervention group and the case–control group. We contacted the corresponding authors when the univariate or multivariate HR was not reported. Studies were excluded if additional information could not be provided.

### 2.6. Statistical Analysis

OR or case data from the intervention and control groups were used to analyse dichotomous data. The heterogeneity among studies was assessed using the Cochrane Q test and I^2^ test. When I^2^ > 30% or *p* < 0.05 indicated considerable heterogeneity, sensitivity and subgroup analyses were performed to determine the effect of each study on the overall outcome. A random effects model was used to obtain pooled estimates. A funnel plot and Egger’s test were used to assess potential publication bias. There are various analytical methods for estimating the accuracy of the comprehensive tests. In this study, cumulative receiver operating characteristic curve (SROC) analysis was used, also known as SROC analysis. Statistical analysis was performed using Stata software version 16 (StataCorp LLC, 4905 Lakeway Drive, College Station, TX, USA), and *p* < 0.05 was considered statistically significant.

## 3. Results

### 3.1. Search Results and Study Characteristics

According to our designated retrieval strategy (Figure 1), 318 records were identified after removing duplicates. Based on the title and abstract screening, we excluded 287 articles. There were 31 articles that were chosen to have their full texts and perused, and nine studies were eventually included in our systematic review and meta-analysis. The studies included in this systematic review were published in nine articles between 2005 and 2020. Seven articles were randomised controlled trials [21,22,23,24,25,26,27], and two were prospective study designs [28,29]. A total of 1049 patients who had undergone PCI and who were stented were included. The mean age was 59.4 ± 9.5(years). There were 185 patients who were diagnosed with ISR during follow-up (1–12 months). All of the investigators completed baseline hs-CRP measurements during the perioperative period of PCI. Table 1 summarises the patient and study characteristics. Methodological assessments and quality assessments of each included article can be found in Supplementary Figures S1 and S2.

### 3.2. The Risk of Higher Hs-CRP to ISR

**Figure 2:**
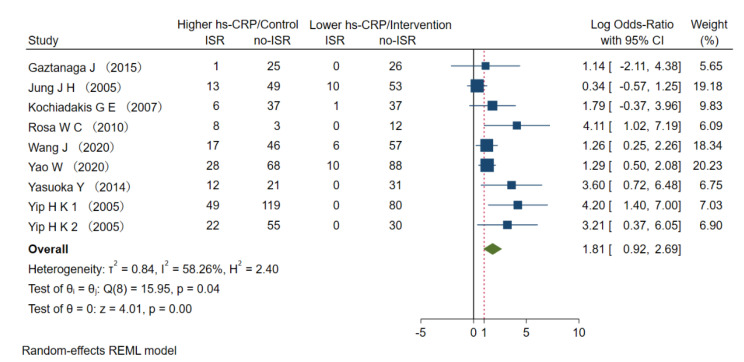
Meta analysis of ORs in studies evaluating the prognostic value of hs-CRP to restenosis in higher vs lower hs-CRP group. OR: odds ratio, hs-CRP: high-sensitive C-reactive protein, ISR: in-stent restenosis [21,22,23,24,25,26,27,28,29].

All of the studies described the perioperative hs-CRP levels. We performed a random-effects model for the meta-analysis and found that higher baseline hs-CRP levels in patients with PCI stents were not significantly associated with increased ISR risk, with OR =1.81 (0.92–2.96) (Figure 2) and I^2^ = 58.26% (*p* < 0.001), indicating some heterogeneity among the studies (Supplemental Figure S3).

### 3.3. Summary Receiver Operating Characteristic Curve

**Figure 3:**
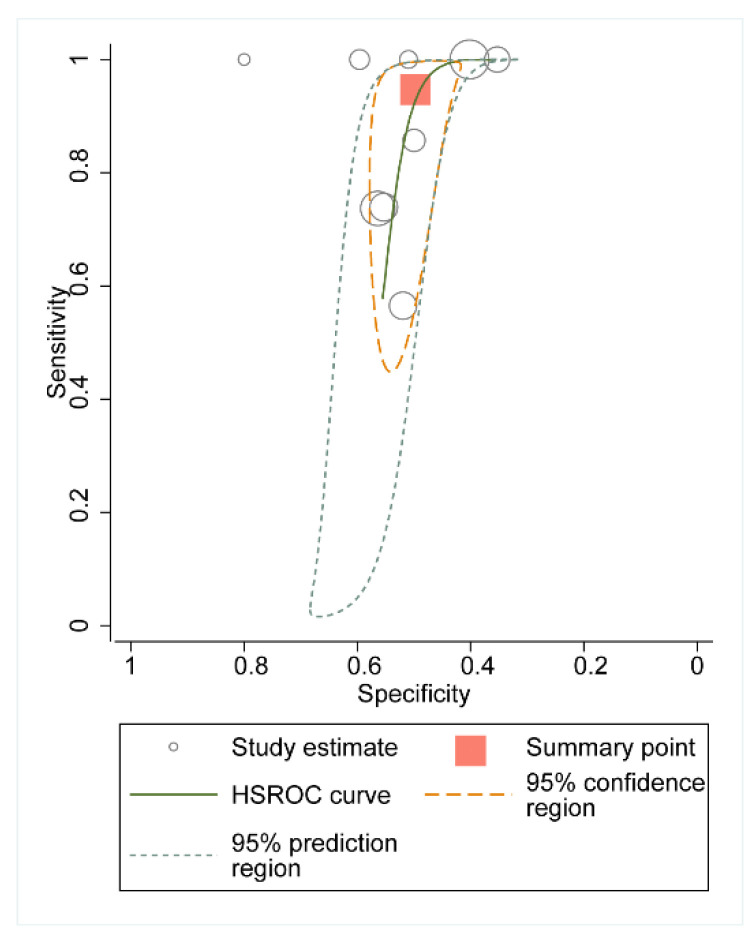
HSROC of the hs-CRP for predicting in-stent restenosis.

The aim of this meta-analysis was to evaluate the diagnostic predictive value of hs-CRP for ISR, so we plotted the HSROC for the total pooled volume of each study (Figure 3). The overall sensitivity of the baseline hs-CRP was 0.95 (0.71–0.99), the specificity was 0.50 (0.45–0.5), the area under the ROC was 0.58 (0.53–0.62), and the diagnostic OR value was 18 (3–116). There was also significant heterogeneity in the diagnostic sensitivity specificity of each trial (Q = 33.88, df = 2.00, *p* < 0.001).

### 3.4. Sensitivity Analysis

**Figure 4:**
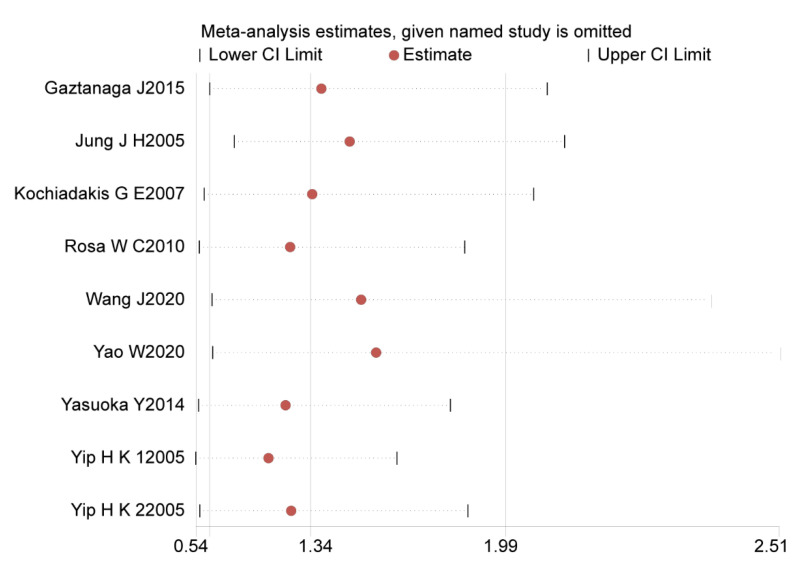
Sensitivity analysis of current meta-analysis [21,22,23,24,25,26,27,28,29].

We eliminated nine studies from sensitivity analysis step-by-step. No individual study significantly affected the pooled effect size, indicating that the sensitivity of this meta-analysis is low and that the results are reliable (Figure 4).

### 3.5. Subgroup Analyses

**Figure 5:**
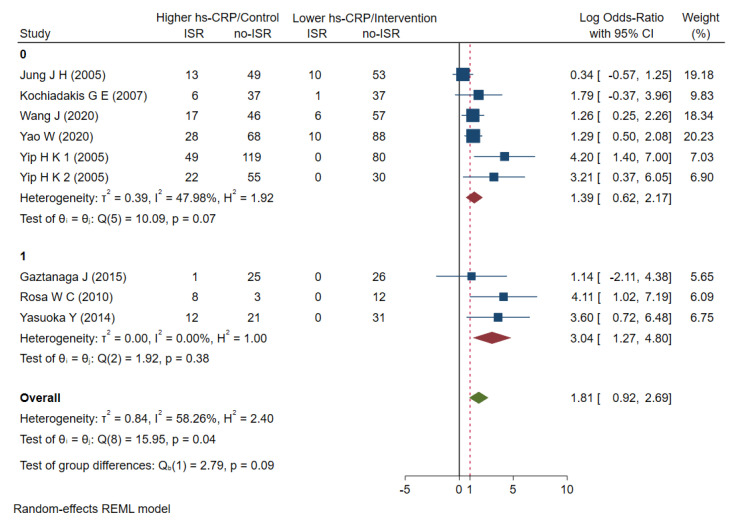
Meta-analysis of ORs in studies evaluating the prognostic value of hs-CRP to restenosis in higher vs lower hs-CRP group as sub-grouped by age. OR: odds ratio, hs-CRP: high-sensitive C-reactive protein, ISR: in-stent restenosis, 0: <60(years), 1: ≥60(years) [21,22,23,24,25,26,27,28,29].

**Figure 6:**
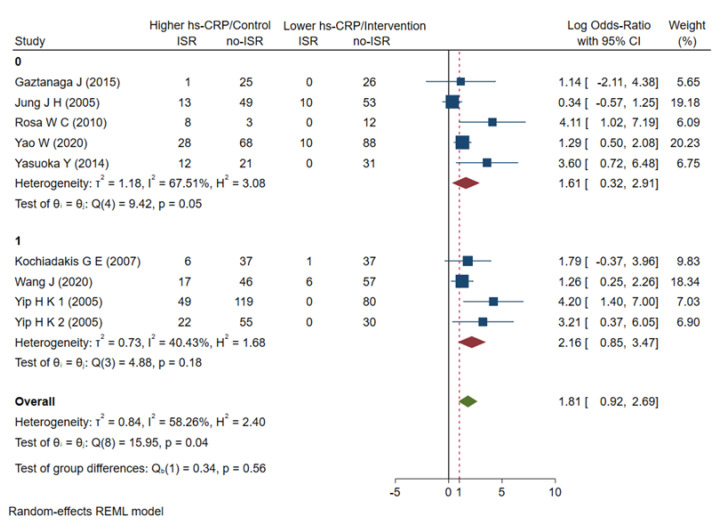
Meta-analysis of ORs in studies evaluating the prognostic value of hs-CRP to restenosis in higher vs lower hs-CRP group as sub-grouped by hs-CRP detecting timepoint. OR: odds ratio, hs-CRP: high-sensitive C-reactive protein, ISR: in-stent restenosis, 0: <6 (months), 1: ≥6 (months) [21,22,23,24,25,26,27,28,29].

**Figure 7:**
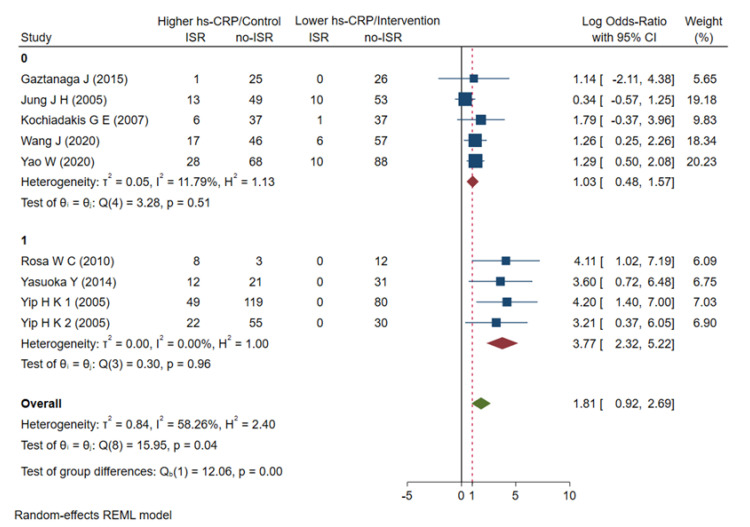
Meta-analysis of ORs in studies evaluating prognostic value of hs-CRP to restenosis in higher vs lower hs-CRP group as sub-grouped by diabetes. OR: odds ratio, hs-CRP: high-sensitive C-reactive protein, ISR: in-stent restenosis, 0: without diabetes 1: complicated with diabetes [21,22,23,24,25,26,27,28,29].

First, we conducted a subgroup analysis according to age, and the results showed that the OR = 2.16 (0.85–3.47) in the ≥60-year-old group and the OR = 1.61 (0.32–2.91) in the <60-year-old group failed to show a significant correlation with ISR risk (Figure 5). A subgroup analysis for age could not sufficiently explain this heterogeneity. Nevertheless, the elderly patient subgroup (≥60-year-old) had a higher risk of ISR compared to the <60-year-old subgroup. We then performed subgroup analyses based on hs-CRP levels at baseline or at admission and at follow-up over the course of 6 months. The hs-CRP level during the follow-up period was significantly associated with an increased risk of ISR events, with OR = 3.04 (1.27–4.80), while the baseline hs-CRP level at admission was not significantly associated with the risk of ISR events, with OR = 1.39 (0.62–2.17) (Figure 6). The hs-CRP levels at different follow-up times partially reveal the heterogeneity in the current meta-analysis and also indicate that hs-CRP can have a higher predictive value at longer follow-up times. Finally, we conducted a subgroup analysis according to whether the ISR patients had diabetes or not. The results showed that the hs-CRP level in the diabetes subgroup was significantly associated with an increased risk of ISR events, with OR = 3.77 (2.32–5.22), while the hs-CRP level in the non-diabetic group had no significant correlation with the risk of ISR events, with OR = 1.03 (0.48–1.57) (Figure 7). The results of the subgroup analysis showed that the hs-CRP levels observed after stent implantation (≥6 months) were more effective in predicting the risk of ISR and were consistent after adjusting for two established ISR risk factors (age and diabetes).

### 3.6. Publication Bias

**Figure 8:**
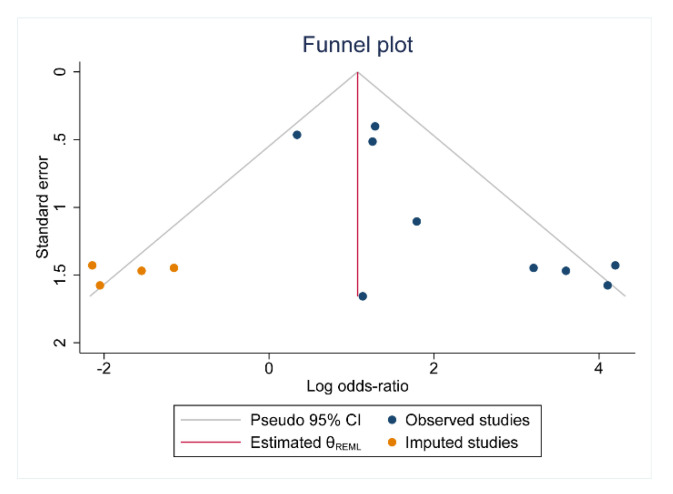
Funnel plot for prognostic value of hs-CRP in in-stent restenosis.

We assessed publication bias by drawing funnel plots and using Egger’s test. Both Egger’s test (Supplementary Figure S4) and the funnel plots (Figure 8) suggest the presence of publication bias in some of the analyses.

## 4. Discussion

Hs-CRP is a marker of oxidative stress and has long been used as a marker of inflammatory diseases. Inflammation is a major determinant of both experimental and clinical in-stent restenosis [30]. It is also an indicator that is used to evaluate the risk of residual inflammation in cardiovascular disease [13]. Therefore, it is suitable to define high-inflammation-risk groups and to predict the occurrence of ISR in the post-stent population. Through early detection and intervention, mortality can be reduced, and medical resources can be saved. Many inflammatory markers have been found to be risk factors for ISR [31], but hs-CRP is currently the most widely used and conveniently detected inflammatory marker in clinical practice. Substantial evidence implicates hs-CRP as a risk factor for ASCVD [32,33]. Hoshida et al. reported that persistently elevated hs-CRP was associated with a risk of restenosis in patients with stable angina but not receiving statin therapy. Gaspardone et al. [34] pointed out that inflammation plays an important role in PCI complications. He explained that this is related to damage to the artery after either balloon expansion or after stenting. In addition, a variety of inflammatory factors, such as cytokines and chemokines, are involved in the neointimal tissue response at the site of coronary stenting [34]. A previous meta-analysis suggested that high baseline hs-CRP levels in patients following stent implantation were associated with an increased risk of ISR [19], but it did not indicate whether long-term ISR, including after 6, 8, and 12 months, could be predicted. At the same time, inflammation is an important pathological basis for the pathogenesis of ISR, so reducing inflammation through drugs can theoretically improve the prognosis of PCI. Another meta-analysis evaluating statin reduction in hs-CRP further suggested that statin therapy can reduce inflammation, reduce hs-CRP levels, and, ultimately, improve prognosis [35]. However, intensive lipid-lowering therapy for ISR patients during PCI did not reduce the risk of ISR [36]. Meanwhile, simvastatin-eluting stents or oral anti-inflammatory drugs have not shown a decrease in the risk of ISR along while also lowering hs-CRP [37,38]. Then, by pooling the results of different studies in a meta-analysis, it was possible to prove the diagnostic value of hs-CRP. 

Our study does not support the findings of the previous meta-analysis [19]. The current meta-analysis and systematic review revealed that baseline hs-CRP levels in the perioperative period of PCI, including before the PCI and within 1 month after stenting, did not accurately predict long-term restenosis events, but the HSROC curves that were comprehensively constructed from the nine trials showed that the model with hs-CRP as the only diagnostic factor still had high sensitivity, which further supports the inflammation pathology in ISR progress. It is interesting that the hs-CRP levels during follow-up (≥6 month) were substantially related to an elevated risk of ISR after we adjusted for age, follow-up time, and diabetes.

It was previously reported that ISR could result from inflammation [30]. The systematic reviews of cohort studies [19,39] have reported many inflammatory biomarkers that are easily available, including IL-6, TNF-γ, MCP-1, CD11b, CRP, and hs-CRP, and that have been found to predict the risk of restenosis. On the other hand, there are contracted findings showing that hs-CRP does not predict restenosis in PCI patients, although hs-CRP is significantly increased in these patients at baseline levels [28,29]. In addition, Kocas et al. suggested that statin nonadherence at follow-up in patients with PCI was associated with an increased risk of ISR [40]. The current meta-analysis supports a chronic inflammatory state compared to acute-phase inflammation when undergoing PCI, which can predict the risk of ISR more effectively. Combined information regarding multiple inflammatory biomarkers at follow-up rather than at baseline could be useful to evaluate the risk of ISR in patients who have undergone PCI.

As reviewed in the current meta-analysis, the role of inflammation in the development of restenosis provides opportunities for therapeutic intervention using anti-inflammatory treatments. In patients presenting systemic evidence of inflammation as measured by an elevated hs-CRP level at baseline and follow-up, the administration of oral anti-inflammatory therapy post-stenting dramatically reduces the rate of restenosis, as reported by Gaztanaga J [21], Rosa W C [22], and Yao W [23]. Meanwhile, Jung J H [24], Kochiadakis G E [25], and Wang J [26] reported that the profound impact of DES on restenosis prevention reflects both its properties as an anti-proliferative as well as an anti-inflammatory agent. Such studies underscore the important role of anti-inflammation in the attendant risks of restenosis or thrombosis. However, heterogeneity was observed in the changes in the inflammatory biomarkers accompanying the effectiveness of anti-inflammatory treatments [27]. Hon-Kan Yip [28,29] et al. reported the hs-CRP trends at baseline, 21 days, 3 months, and 6 months in patients with unstable angina pectoris. Nonetheless, these trends were not useful for predicting restenosis after coronary stenting. As an indicator of chronic inflammation, Hs-CRP is not alone and is accompanied by diabetes and age. Combined information regarding multiple risk factors at follow-up rather than admission could be useful for evaluating the risk of ISR in patients who have undergone PCI. Additionally, with advancements in interventional technology, whether this long-term anti-inflammatory treatment is necessary is a point of controversy. The short-term release of anti-inflammatory agents from drug-coated balloons can effectively block the inflammatory cascade and the occurrence of persistent chronic inflammation [41]. In addition, the drug-intensive anti-inflammatory efficacy was better during hospitalisation than during follow-up [40]. Therefore, the acute-phase inflammation can be effectively controlled, and the inflammatory biomarker in the follow-up period can better reflect the lesion’s progress.

The current meta-analysis shows a prognostic value of hs-CRP for ISR and that baseline hs-CRP levels cannot define ISR in high-risk patients. More investigations may be required in the future to determine the utility of hs-CRP during follow-up. At the same time, risk factors for ISR management are becoming more well-recognised. It will be interesting to construct a comprehensive model based on the numerous biomarkers and risk factors that may increase the predictive value of hs-CRP.

There are a few limitations to our research. First, our study seems to have publication bias, which could be due to the fact that positive outcomes are easier to report and publish. Second, we were unable to describe the effects of different interventions on hs-CRP in the meta-analysis due to a lack of data.

## 5. Conclusions

Overall, the current meta-analysis demonstrates that the hs-CRP levels at follow-up, rather than those at baseline, are related to higher ISR risk, indicating that they could be used as a prognostic marker during long-term follow-up. After adjusting for diabetes, age, and follow-up time, this hazard was higher.

## Figures and Tables

**Table 1:** Basic characteristics of include studies. N/A: not available.

Study	Year	Country/Area	Design	Sample size	Age (Years)	Higher hs-CRP ISR/n	Higher hs-CRP non-ISR/n	Lower hs-CRP ISR/n	Lower hs-CRP non-ISR/n	Follow-Up (Months)	Comparision	Time for Test
Gaztanaga, J. [21]	2015	USA	RCT	52	56.6 ± 9.67	1	25	0	26	6	Higher hs-CRP vs. Lower hs-CRP 5-lipoxygenase inhibitor vs. placebo	0
Jung, J.H. [24]	2005	Korea	RCT	125	59 ± 10.0	13	49	10	53	6	Restenosis vs. non-restenosis Carbon-implanted surface stents vs. Control stents	0/48 h/6 m
Kochiadakis, G.E. [25]	2007	Greece	RCT	81	62 ± 11.0	6	37	1	37	1	Higher hs-CRP vs. Lower hs-CRP Bare Metal Stents vs. Sirolimus-eluting Stents	0/24 h/48 h/6 m
Rosa, W.C. [22]	2010	Brasil	RCT	48	56.8 ± 13	8	3	0	12	2	Restenosis vs. non-restenosis Oral sirolimus vs. Placebo	0/24 h/7 d/49 d/2 m
Wang, J. [26]	2020	China	RCT	126	66.93 ± 5.25	17	46	6	57	12	Restenosis vs. non-restenosis Rapamycin-eluting double stenting vs. single stenting	0/3 m
Yao, W. [23]	2020	China	RCT	194	55.1 ± 8.3	28	68	10	88	6	Restenosis vs. non-restenosis Valsartan vs. control	0/6 m
Yasuoka, Y. [27]	2014	Japan	RCT	68	N/A	12	21	0	31	6	Restenosis vs. non-restenosis Bare Metal Stents vs. Control	0/6 m
Yip, H.K. [28]	2005	China	Cohort	248	61.3 ± 9.4	49	119	0	80	6	Restenosis vs. non-restenosis Unstable angina vs. Risk control and Normal control	0/21 d/3 m/6 m
Yip, H.K. [29]	2005	China	Cohort	107	61.0 ± 10.3	22	55	0	30	7	Restenosis vs. non-restenosis Unstable angina vs. Normal control	0/21 d/3 m/7 m

## Data Availability

The original contributions presented in the study are included in the article/Supplementary Materials, further inquiries can be directed to the corresponding authors.

## Supplementary Materials

The following supporting information can be downloaded at: https://www.mdpi.com/article/10.3390/jcdd9080247/s1, Supplementary Figures S1 Risk of bias and applicability concerns: reviewers’ judgments about each domain presented as percentages across included studies. Supplementary Figures S2 Risk of bias and applicability concerns: reviewers’ judgments about each domain for each included study. Supplementary Figures S3 L’Abbé plot for current meta-analysis. Supplementary Figures S4 Egger’s plot of current meta-analysis.
