# Supplementary material for: Prognostic Value of High-Sensitivity C-Reactive Protein in In-Stent Restenosis: A Meta-Analysis of Clinical Trials
Source: J Cardiovasc Dev Dis. 2022 Aug 4;9(8):247. doi: 10.3390/jcdd9080247 (PMC9409410; doi:10.3390/jcdd9080247)
Supplement: Supplementary file 1 [file jcdd-09-00247-s001.zip › jcdd-1800793-supplementary.pdf]

Journal of
Cardiovascular Development and Disease
MDPI

Supplement Figures
Figure legends

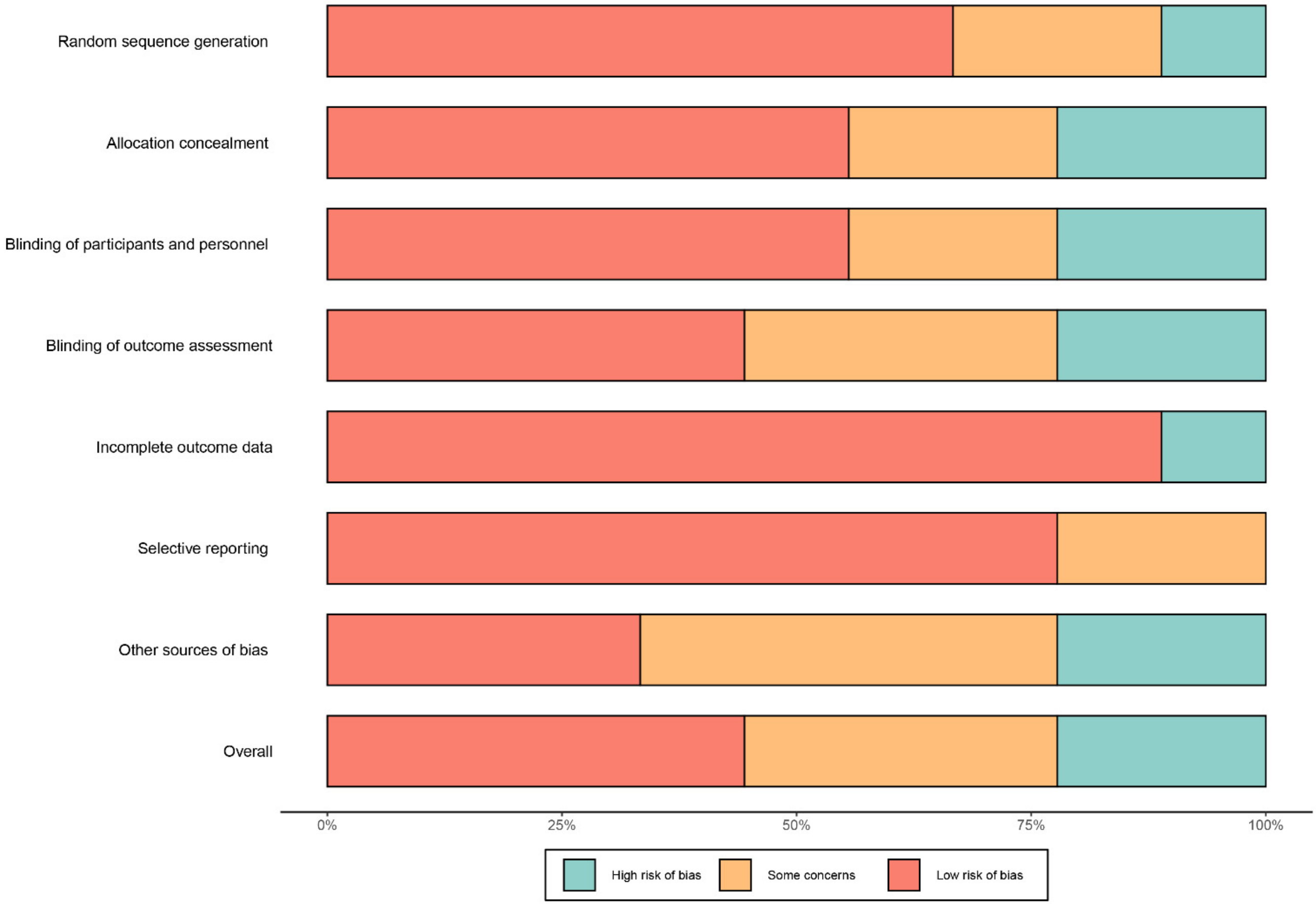

Supplementary Figure S1 Risk of bias and applicability concerns: reviewers' judgments about each domain presented as percentages across included studies.

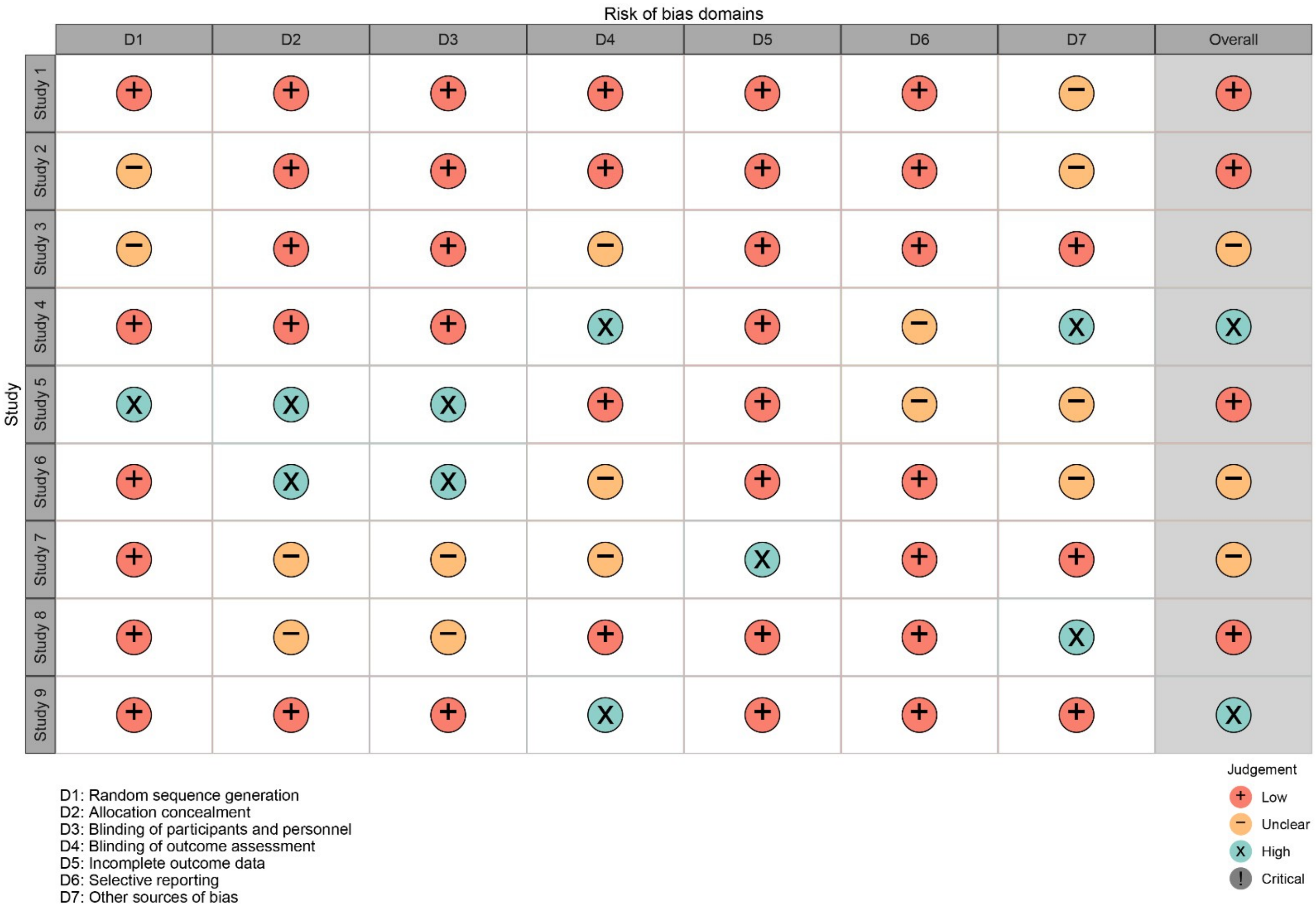

Supplementary Figure S2 Risk of bias and applicability concerns: reviewers' judgments about each domain for each included study.

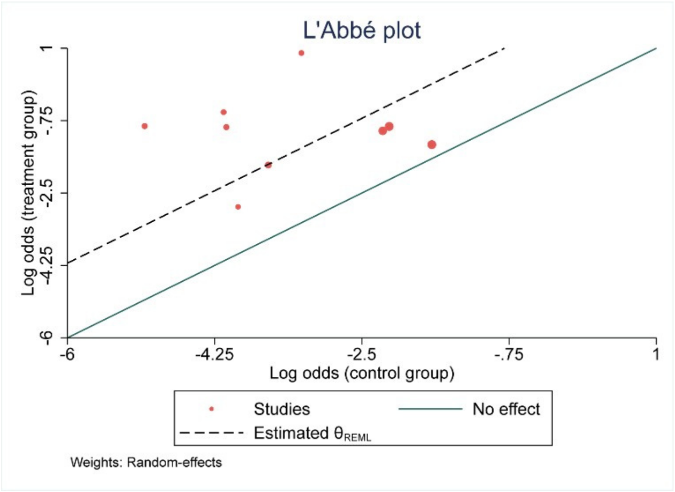

Supplementary Figure S3 L'Abbéplot for current meta -analysis.

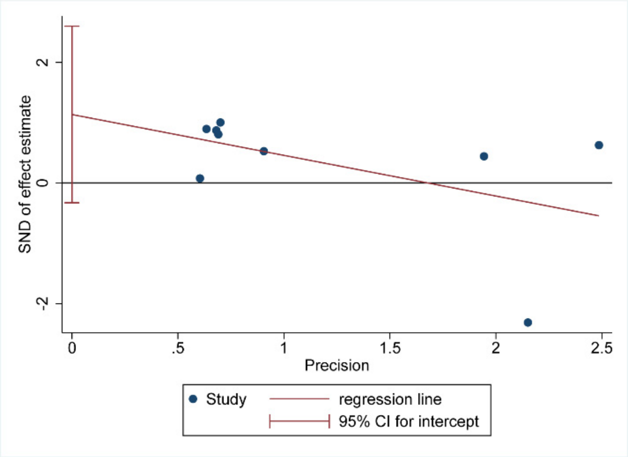

Supplementary Figure S4 Egger's plot of current meta-analysis.
